# Prognostic role of pretreatment neutrophil to lymphocyte ratio in breast cancer patients: A meta-analysis: Erratum

**DOI:** 10.1097/MD.0000000000009727

**Published:** 2018-01-26

In the article, “Prognostic role of pretreatment neutrophil to lymphocyte ratio in breast cancer patients: A meta-analysis”,^[1]^ which appeared in Volume 96, Issue 45 of *Medicine*, two authors’ affiliations were reversed.

Dr. Hang-Yin Qu's affiliation should be The Department of Oncological Surgery, Shaanxi University of Chinese Medicine, Shaanxi, P.R. China.

Dr. Xiao-Yi Duan's affiliation should be The Department of Radiology, the First Affiliated Hospital of Xi’an Jiaotong University, Xi’an, Shaanxi, P.R. China.
